# A mechanism-guided simulation framework for synthetic tax-risk data: Constraint validation and reproducible benchmarking

**DOI:** 10.1371/journal.pone.0353137

**Published:** 2026-10-01

**Authors:** Xiaojing Fan

**Affiliations:** Business School, University of Shanghai for Science and Technology, Shanghai, China; Liverpool John Moores University, UNITED KINGDOM OF GREAT BRITAIN AND NORTHERN IRELAND

## Abstract

Synthetic benchmarks can support tax-risk method development when administrative records are inaccessible, but a simulator can also predetermine the patterns later reported as detection findings. We present the Tax Data Generation Model (TDGM), a mechanism-guided simulator that enforces specified income-statement and balance-sheet identities, generates a three-year panel for 10,000 firms, and injects four prespecified risk strategies. This study uses no administrative audit data; its purpose is to test whether the implementation reproduces its design assumptions and to provide a reproducible benchmark. All predictive evaluations used five repeated 70/30 group splits by firm, so no firm appeared in both training and test data. Observed marginal effects were heterogeneous rather than uniformly small: for all risky observations, Cohen’s d was −1.29 for profit margin, −1.20 for tax burden, 1.05 for the transfer-pricing indicator, and 0.15 for the income-gap ratio. In a controlled cross-sectional comparison, raw CTGAN failed both row-level accounting identities, whereas deterministic projection restored 100% validity but worsened dependence fidelity. Under the revised classifier specification, a one-hidden-layer neural network with conventional L2 regularization achieved mean ROC-AUC 0.9942 and PR-AUC 0.9710; XGBoost achieved 0.9714 and 0.8975, respectively. Removing the income-gap ratio changed XGBoost ROC-AUC only from 0.9714 to 0.9702, while restricting the model to raw Level 1 variables reduced it to 0.8941. At 1% prevalence, XGBoost ROC-AUC remained 0.9720 but PR-AUC fell to 0.6703, showing why prevalence-sensitive metrics are required. Together, the three contributions are a constraint-by-construction simulator, a reusable audit protocol for mechanism-guided generators, and explicit measurement of the accounting-repair/statistical-fidelity trade-off. Because selected flows are changed after stock generation, derived ratios can carry design-induced information. These are internal properties of a synthetic data-generating process, not estimates of real tax-evasion behavior or deployable audit performance.

## 1. Introduction

Tax non-compliance creates a substantial fiscal enforcement problem, while firm-level audit records are rarely available for open methodological research because of tax-secrecy and confidentiality rules [1,2]. Synthetic data can therefore be useful for testing computational workflows, provided that the limits of simulation-based evidence are explicit.

Machine-learning models have been used to rank cases for tax and customs audits, and recent studies already evaluate nonlinear classifiers with lift or top-budget metrics [3–7]. The detection component of this study is therefore not presented as evidence that gradient boosting or top-k evaluation is novel. Its role is to probe whether a transparent synthetic generator produces the intended—and unintended—predictive structure.

Synthetic tabular-data methods include multiple imputation, conditional generative adversarial networks, diffusion models, and domain-specific financial-data applications [8–12]. These methods learn distributional structure, whereas accounting identities are hard row-level constraints. A general-purpose generator can be combined with post-sampling projection, but projection may alter marginal and dependence fidelity; a matched comparison should disclose that trade-off rather than treating exact constraint validity as a universal measure of generative quality.

Accordingly, TDGM is framed as a constraint-by-construction simulator and benchmark, not as a general replacement for learned synthetic-data models. The relevant validation questions are whether its code implements the documented rules, whether those rules create identifiable artifacts, how results change under feature ablation and grouped validation, and how a transparent CTGAN repair baseline behaves under the same cross-sectional inputs.

Risk behavior in the simulator is heterogeneous. Revenue suppression is restricted to 2%–5%, but cost inflation uses 25%–40%, transfer manipulation shifts 20%–35% of profit, and shell companies are intentionally overt stress tests. The revised analysis therefore reports observed effect sizes and per-strategy performance instead of describing all risky cases as a single low-signal class.

We implement TDGM as an auditable data-generating process with fixed accounting identities, a Pareto firm-size distribution, AR(1) revenue dynamics, a Markov risk state, and documented strategy-specific manipulations. Audit effects can persist over time in real settings [13]. However, the transition probabilities used here are scenario parameters rather than empirical estimates. The generated data are then analyzed with firm-level train/test separation, repeated splits, PR-AUC, calibration measures, feature ablations, and strategy-specific results.

This paper makes three bounded contributions.

(1) We provide a transparent mechanism-guided simulator that enforces specified accounting identities and exposes its parameters, assumptions, and unused legacy fields.
(2) We formulate a reusable audit protocol for mechanism-guided synthetic generators, combining parameter traceability, row-level constraint verification, mechanism-specific realized effect sizes, entity-level leakage control, feature ablation, prevalence-sensitive discrimination and calibration, noise stress testing, and matched baseline comparisons with transparent constraint repair.
(3) We provide a reproducible, like-for-like cross-sectional CTGAN comparison with a deterministic accounting-projection variant, thereby separating constraint validity from distributional fidelity and documenting the cost of repair.

## 2. Literature review

### 2.1. Tax-risk simulation and signal strength

Large-scale audit experiments indicate that non-compliance can concentrate in self-reported income that is not covered by third-party reporting [14]. Threshold-monitoring research also documents strategic bunching of reported revenue around enforcement cutoffs [15]. These studies motivate heterogeneous simulation scenarios but do not calibrate the magnitudes used in TDGM.

Observed tax behavior can be strategic and clustered around enforcement rules [14,15]. However, the present simulator does not claim to estimate the signal strengths of real evasion. Instead, its manipulation ranges are scenario assumptions that must be evaluated from the realized dataset. This distinction is important because different strategies in the current code generate very different marginal effects, including several large effects.

### 2.2. Machine learning for audit targeting and the benchmark gap

Comparisons between logistic regression and flexible machine-learning models depend on data structure, tuning, and validation design [16–18]. Statistical fraud detection and anomaly detection also provide established alternatives, including isolation-based methods [19–21]. In tax enforcement, gradient boosting, network features, and audit-budget ranking metrics are already established. Baghdasaryan et al. evaluated tax-fraud detection using top-decile lift; Battaglini et al. studied machine-learning allocation of tax audits; Alwanin et al. used gradient boosting for customs risk ranking; and Borrotti et al. used public accounting information and random forests to predict aggressive tax-location decisions by European groups [4–7].

The gap addressed here is narrower: few openly reproducible tax-risk benchmarks make accounting constraints, longitudinal assumptions, manipulation mechanisms, and leakage controls simultaneously inspectable. TDGM is offered as one such simulation test bed. Its predictive outputs are diagnostic properties of the generator and must not be read as field evidence about enforcement effectiveness.

## 3. Design rationale and validation questions

### 3.1. Concealed and overt simulated signals

Let P0 and P1 denote the simulated feature distributions for compliant and risky observations. In a mechanism-guided benchmark, their separation is determined by the chosen rules. Marginal overlap and multivariate separability are therefore design targets to be measured after generation, not assumed empirical facts about tax evasion.

TDGM combines one deliberately subtle manipulation (2%–5% revenue suppression) with larger cost and transfer manipulations and an overt shell-company stress test. Fig 1 visualizes the supplied 30,000-observation synthetic panel. Table 1 and Fig 4 report the resulting marginal effect sizes, which show that the original universal d<0.2 statement was incorrect.

**Table 1:** Observed marginal Cohen’s d for all risky versus compliant observations.

Level	Feature	Cohen’s d	Magnitude
Level 1	revenue	−0.018	negligible
Level 1	cost	0.012	negligible
Level 1	profit	−0.068	negligible
Level 1	tax	−0.068	negligible
Level 1	asset	0.157	negligible
Level 1	liability	−0.022	negligible
Level 1	equity	0.165	negligible
Level 1	years_in_operation	0.022	negligible
Level 1	profit_margin	−1.294	large
Level 1	tax_burden	−1.198	large
Level 1	asset_turnover	−0.009	negligible
Level 2	vat_sales	−0.014	negligible
Level 2	income_gap_ratio	0.150	negligible
Level 2	high_risk_invoice_ratio	−0.300	small
Level 2	related_party_revenue_ratio	0.272	small
Level 2	transfer_pricing_anomaly	1.045	large
Level 2	electricity_consumption	−0.035	negligible
Level 2	operating_expenses	−0.023	negligible
Level 2	rd_expenses_ratio	−0.017	negligible

**Fig 1:**
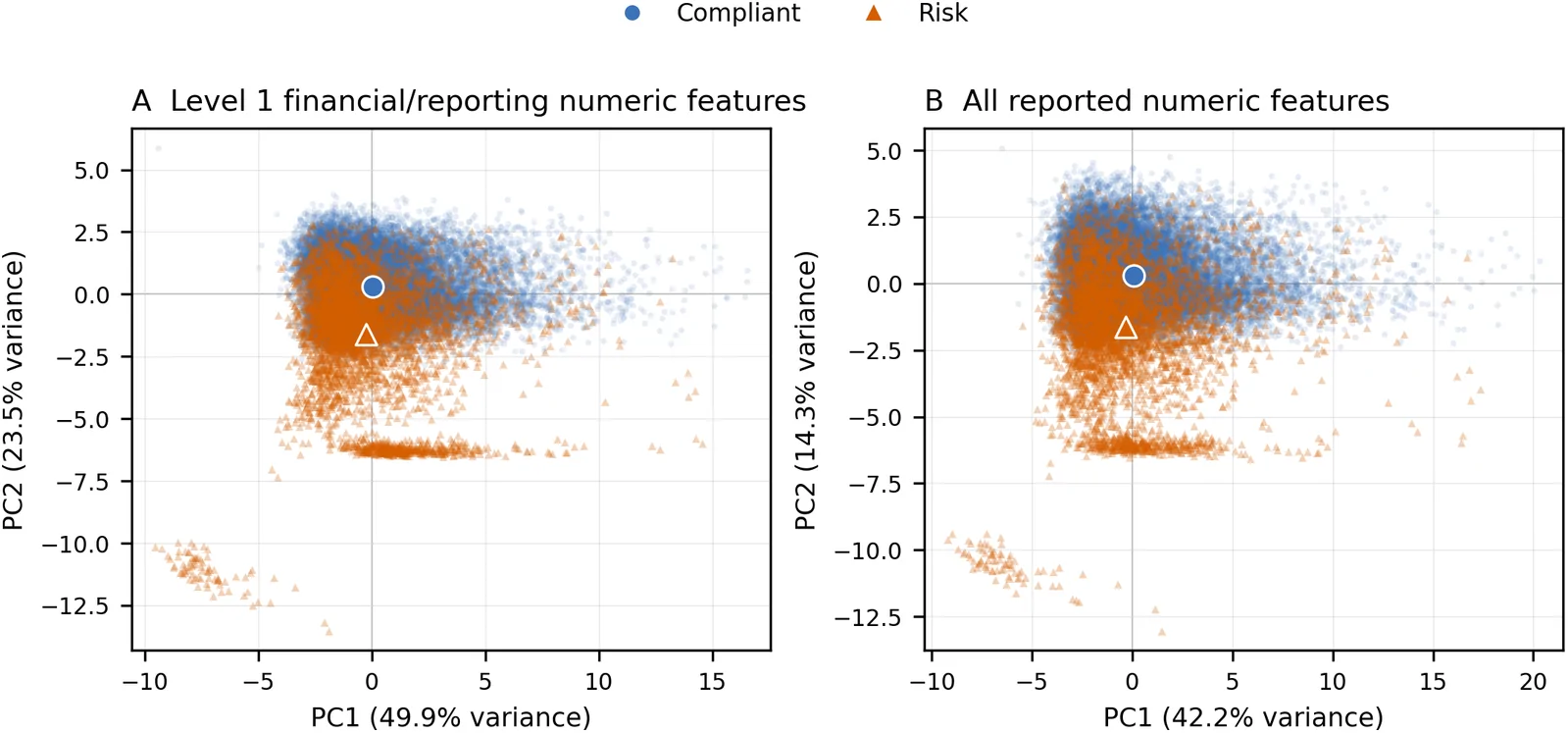
Principal-component projections of the supplied synthetic TDGM panel. Panel A uses Level 1 financial/reporting numeric features and Panel B uses all reported numeric features after signed-log transformation of monetary quantities and standardization. Points represent the 30,000 generated firm-year observations; colors are used only for visualization and were not included as features. The first two components explain 73.37% of Level 1 variance and 56.46% of all-reported-numeric variance. The overlap is descriptive of this synthetic dataset and does not establish real-world concealment.

### 3.2. Mechanism-guided logic and prespecified validation checks

TDGM contains three programmed layers and one induced feature relationship. We state them as validation checks so that later results are interpreted as implementation tests rather than independent discoveries.

#### 3.2.1. Accounting consistency (Validation Check 1).

Accounting identities form the foundation of financial statements. Violating them makes synthetic data unsuitable for tax risk modeling.

Layer 1 (Accounting Logic): We build financial rules directly into the system. Every sample must satisfy the identity:


$$\begin{array}{c} \text{Assets }\equiv \text{ Liabilities }+\text{ Equity} \end{array}$$ (1)

Validation Check 1: Specified Identities. Equity is computed as assets minus liabilities, and profit is computed as revenue minus cost. The implementation should therefore satisfy the two documented identities within numerical tolerance for every TDGM row.

#### 3.2.2. Strategy-specific signal strength (Validation Check 2).

TDGM injects a heterogeneous mixture of simulated risk mechanisms: revenue suppression is comparatively subtle, cost inflation and transfer manipulation create stronger signals, and the shell-company rule is an overt stress test.

Layer 2 (risk injection): manipulation magnitudes are strategy-specific—2%–5% of revenue for revenue suppression, 25%–40% of cost for cost inflation, 20%–35% of profit for transfer manipulation, and an overt shell-company stress test.

Validation Check 2: Realized Separation. The generated dataset should be inspected using per-feature Cohen’s d calculated with the pooled standard deviation and per-strategy prediction metrics [22]. No universal d<0.2 condition is imposed on all strategies, and nonlinear-model performance is treated as a property of this benchmark.

#### 3.2.3. Programmed temporal persistence (Validation Check 3).

Firm performance exhibits persistence over time.

Layer 3 (Dynamic Evolution): We link current revenue to past values using an AR(1) process:


$$\begin{array}{c} \text{ln}R_{i,t}^{\text{true}}=\rho \text{ln}R_{i,t-\text{1}}^{\text{true}}+(\text{1}-\rho )\mu_{i}+\epsilon_{i,t},\;\rho =\text{0}.\text{85},\;\sigma_{\epsilon }=\text{0}.\text{12} \end{array}$$ (2)

where $\epsilon_{i,t}\sim N\left(\text{0},\sigma_{\epsilon }^{\text{2}}\right)$ are i.i.d. shocks, and $\mu_{i}$ is the firm-level long-run mean.

Validation Check 3: Programmed Persistence. Revenue follows the AR(1) equation with ρ=0.85 and $\sigma_{\varepsilon }$=0.12. Because the firm-specific long-run mean is known only inside the simulator, centered persistence estimates are implementation checks and do not provide a transferable estimator for real panels.

#### 3.2.4. Flow–stock feature construction (Validation Check 4).

The programmed generation order induces a fourth property: for Strategies 1–3, selected flow variables are changed after stock variables have already been generated. Ratios that combine those quantities can therefore change as a direct consequence of code order.

Validation Check 4: Flow–stock Construction. For Strategies 1–3, selected flow variables are changed after stock variables are generated. Ratios that combine those variables can therefore carry design-induced predictive information. Feature ablations quantify how much performance depends on derived ratios and Level 2 verification variables.

## 4. Materials and methods

TDGM implements a bottom-up simulation in which firm attributes, economic trajectories, accounting quantities, risk states, and observable indicators are generated in sequence. Fig 2 and Algorithm 1 summarize the workflow; the parameter dictionary in S6 Data distinguishes active parameters from legacy fields retained in the original code.

**Fig 2:**
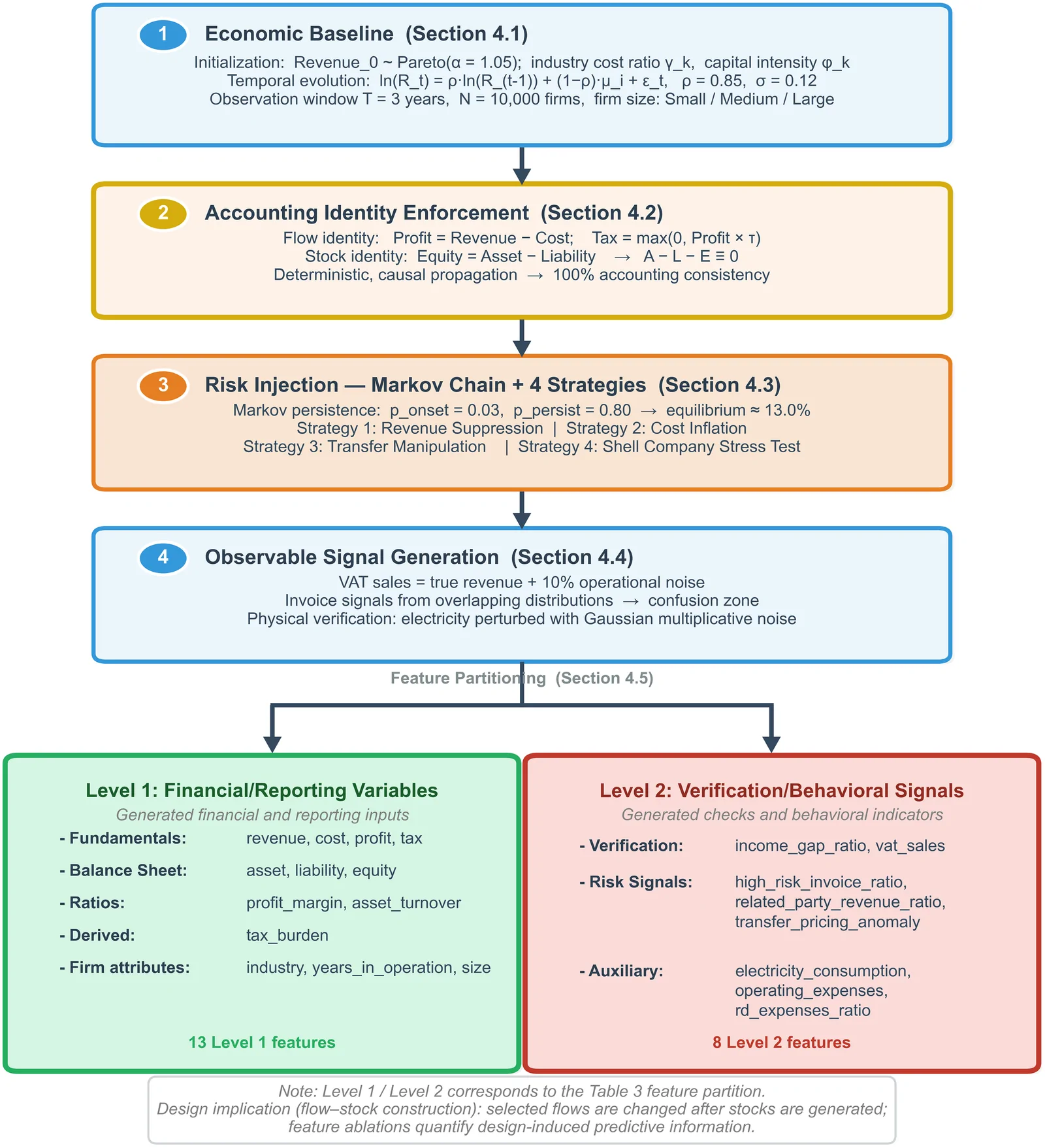
TDGM mechanism-guided simulation architecture.

Algorithm 1: Tax Data Generation Model (TDGM).

Input:

 N = number of firms; T = observation window (default: T = 3)

B = burn-in periods (default: B = 7) ▷ discard first B periods to reduce initialization effects

 AR(1) parameters: ρ = 0.85, σε = 0.12

 Industry parameter sets: {γk, φk}, k = 1,...,K (cost ratio, capital intensity)

 Initial risk by size: Small=0.18, Medium=0.15, Large=0.12

 Markov risk parameters: p_onset = 0.03, p_persist = 0.80

 Evasion magnitude: δ ~ U[0.02, 0.05] (Strategy 1); Seed = 42

Output: Synthetic panel dataset D = {(x_{i,t}, y_{i,t})}, i = 1..N, t = 1..T

Phase 1: Economic Baseline (Section 4.1)

1.  for i = 1 to N do

2.   R_{i,0} ~ Pareto(α = 1.05, x_m = 10^5^) ▷ heavy-tailed firm-size init.

3.   μ_i ← ln R_{i,0}; assign industry k_i

4.   for t = 1 to B + T do

5.    ε_{i,t} ~ N(0, σε²)

6.    ln R_{i,t} ← ρ·ln R_{i,t − 1} + (1 − ρ)·μ_i + ε_{i,t} ▷ AR(1) evolution

if t ≤ B then continue ▷ burn-in: update state but do not record

Phase 2: Accounting Identity Enforcement (Section 4.2)

7.   C_{i,t} ← R_{i,t}·(γ_k + ε_cost), ε_cost ~ N(0, 0.05²)

8.   π_{i,t} ← R_{i,t} − C_{i,t}; T_{i,t} ← max(0, π_{i,t}·τ_eff)

9.   A_{i,t} ← R_{i,t}·(φ_k + ε_asset), ε_asset ~ N(0, 0.2²)

10.   λ_{i,t} = clip(N(0.60, 0.15²), 0.3, 0.8); L_{i,t} ← A_{i,t}·λ_{i,t}

11.   E_{i,t} ← A_{i,t} − L_{i,t} ▷ residual: A ≡ L + E holds by construction

Phase 3: Risk Injection (Section 4.3)

12.   Update risk status s_{i,t} via Markov chain (u ~ U[0,1]):

    s_{i,t} = 1 if s_{i,t − 1}=0 and u ≤ p_onset

    s_{i,t} = 1 if s_{i,t − 1}=1 and u ≤ p_persist

    s_{i,t} = 0 otherwise

13.   if s_{i,t} = 1 then apply strategy-specific manipulation:

    S1 Revenue: R ← R·(1 − δ), δ ~ U[0.02, 0.05] ▷ subtle programmed change

    S2 Cost: C ← C·(1 + δ’), δ’ ~ U[0.25, 0.40] ▷ invoice fraud

    S3 Shifting: C ← C + π·γ, γ ~ U[0.20, 0.35] ▷ Transfer Manipulation

    S4 Shell: A = L×100, C = R×0.95 ▷ overt stress-test strategy

    ▷ Stock variable A unchanged in S1–S3 (anchored to pre-manipulation scale)

Phase 4: Observable Signal Generation (Section 4.4)

14.   V_{i,t} ← R^{true}_{i,t}·(1 + η), η ~ N(0, 0.10²) ▷ VAT from true revenue (3rd-party)

15.   Add strategy-specific invoice ratios, transfer indicator, and noisy electricity/expense signals

16.   Partition features: Level 1 (financial/reporting)/ Level 2 (verification/audit) [Table 2]

17.   y_{i,t} ← s_{i,t}

  end for

end for

Return D

Complexity: O(N·T) time and O(N·T·d) space. Runtime depends on hardware; no cross-hardware speedup claim is made.

### 4.1. Economic baseline: Firm initialization and time dynamics

This stage defines the simulated economic environment. The choices below are modeling assumptions used to create a benchmark; they are not estimates from the present study.

**Table 2:** Feature Partitioning and Definitions.

Level	Category	Feature name	Description	Source
Level 1	Fundamentals	revenue, cost, profit, tax	Reported flow variables	Financial statements
Level 1	Fundamentals	asset, liability, equity	Reported stock variables	Financial statements
Level 1	Ratios	profit_margin, asset_turnover	Derived financial-efficiency ratios	Derived
Level 1	Ratios	tax_burden	Tax relative to revenue	Derived
Level 1	Firm attributes	industry, years_in_operation, size	Simulated firm attributes	Registry/reporting
Level 2	Verification	income_gap_ratio, vat_sales	VAT–reported revenue comparison	Tax system/third party
Level 2	Risk signals	high_risk_invoice_ratio	Share of input invoices from risky suppliers	Invoice system
Level 2	Risk signals	related_party_revenue_ratio	Related-party share of revenue	Disclosure/audit
Level 2	Risk signals	transfer_pricing_anomaly	Simulated arm’s-length deviation indicator	Audit
Level 2	Auxiliary	electricity_consumption	Physical activity proxy	Third party
Level 2	Auxiliary	operating_expenses	Operating-expense quantity	Derived
Level 2	Auxiliary	rd_expenses_ratio	R&D expenditure relative to revenue	Disclosure
Level 2	Auxiliary	other generated verification fields	Additional model inputs documented in S6 Data	Simulated

#### 4.1.1. Firm initialization and time evolution.

The initial firm-revenue distribution is a Pareto scenario intended to generate a heavy-tailed rank-size pattern, as motivated by prior firm-size evidence [23]. It is not calibrated to a particular tax population:


$$\begin{array}{c} R_{i,\text{0}}\sim Pareto\left(\alpha =\text{1}.\text{05},x_{m}={\text{10}}^{\text{5}}\right) \end{array}$$ (3)

This scenario produces a heavy-tailed rank-size pattern with many smaller firms and fewer large firms; Fig 3 checks the implemented Pareto scaling rather than claiming calibration to a particular tax population [23].

**Fig 3:**
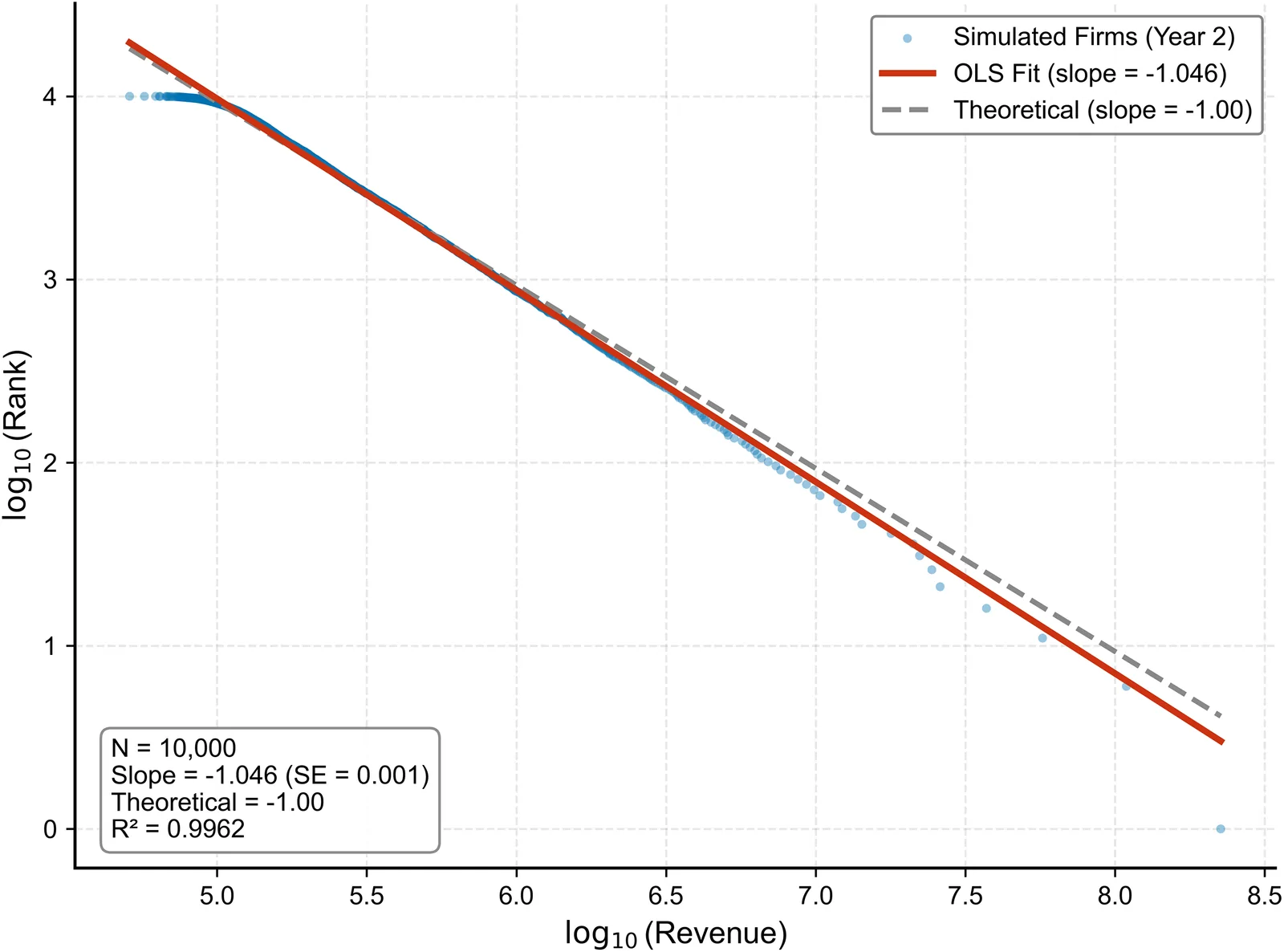
Internal rank-size check against the programmed Pareto scaling.

Firm performance follows equation (2) with ρ=0.85 and innovation standard deviation $\sigma_{\varepsilon }$=0.12. Each firm begins at its programmed long-run mean and undergoes B = 7 unrecorded updates before T = 3 recorded years. Because the long-run mean is known by construction, it can be used for an internal persistence check; this does not eliminate dynamic-panel bias in applications where firm means are unknown [24].

#### 4.1.2. Industry-specific financial structure.

To introduce cross-sectional scenario heterogeneity, TDGM assigns industry-specific parameters governing the simulated financial structure. For firm i in industry k, operating cost and asset scale are determined by the programmed equations below:


$$\begin{array}{c} C_{i,t}=R_{i,t}\times \left(\gamma_{k}+\epsilon_{\text{cost}}\right),\epsilon_{\text{cost}}\sim N\left({\text{0},\text{0}.\text{05}}^{\text{2}}\right) \end{array}$$ (4)

$$\begin{array}{c} A_{i,t}=R_{i,t}\times \left(\phi_{k}+\epsilon_{\text{asset}}\right),\;\epsilon_{\text{asset}}\sim N({\text{0},\text{0}.\text{2}}^{\text{2}}) \end{array}$$ (5)

where $\gamma_{k}$ is the active industry cost ratio and $\varphi_{k}$ is capital intensity. The code also contains legacy target-profit-margin, size-probability, and revenue-range entries that do not affect generation; these are explicitly marked as unused in S6 Data. Table 3 reports the values that determine the generated financial statements.

**Table 3:** Sector-Specific Simulation Parameters ^a^.

Sector	Industry probability	Active cost ratio $\gamma_{k}$	Implied pre-risk margin (1−$\gamma_{k}$)	Capital intensity $\varphi_{k}$
Manufacturing	0.25	0.65	0.35	1.50
Retail	0.20	0.70	0.30	1.20
Services	0.30	0.55	0.45	0.80
Construction	0.15	0.68	0.32	1.30
Technology	0.10	0.50	0.50	1.00

^a^Profit is derived as revenue minus cost; the implied pre-risk margin is therefore approximately 1−$\gamma_{k}$, not the unused legacy target-margin field. Cost noise is Normal(0,0.05²) and capital-intensity noise is Normal(0,0.20²), with the code’s stated clipping rules.

### 4.2. Programmed accounting-constraint construction

TDGM enforces accounting identities through a deterministic flow-to-stock sequence. Unlike the fitted single-table baseline used here, the simulator computes selected variables from previously generated quantities instead of generating all seven financial variables jointly.

(1) Flow Generation

Operational variables are generated first. Revenue and cost are determined by the dynamic process described above, after which profit and tax liabilities are computed as:


$$\begin{array}{c} \pi_{i,t}=R_{i,t}-C_{i,t} \end{array}$$ (6)

$$\begin{array}{c} T_{i,t}=\text{max}\left(\text{0},\pi_{i,t}\times \tau_{\text{effective}}\right) \end{array}$$ (7)

where $\tau_{\text{effective}}$ denotes the simulated effective tax rate.

(2) Stock Derivation

Stock variables are derived from the operational scale of the firm. Total assets $A_{i,t}$ are determined using the capital-intensity parameter $\phi_{k}$ as defined in Equation (5), with revenue serving as the anchor variable. Liabilities $L_{i,t}$ are then generated based on a stochastic debt ratio drawn from $\lambda_{i,t}\sim N\left(\text{0}.\text{6}\text{0},\;{\text{0}.\text{1}\text{5}}^{\text{2}}\right)$, clipped to [0.3,0.8]:


$$\begin{array}{c} L_{i,t}=A_{i,t}\times \lambda_{i,t} \end{array}$$ (8)

(3) Identity Constraint Enforcement

Finally, equity $E_{i,t}$ is defined as the residual term:


$$\begin{array}{c} E_{i,t}=A_{i,t}-L_{i,t} \end{array}$$ (9)

This deterministic construction ensures that the accounting identity holds


$$\begin{array}{c} A_{i,t}-L_{i,t}-E_{i,t}=\text{0} \end{array}$$ (10)

exactly for every generated observation. Because selected flows are modified later for Strategies 1–3, derived ratios may then carry code-order-induced information; the ablation analysis measures the realized effect.

### 4.3. Prespecified simulated risk injection

To create heterogeneous benchmark labels, TDGM uses a two-stage programmed mechanism: risk-state determination followed by conditional strategy selection.

#### 4.3.1. Time Persistence (Markov Process).

The simulated risk state follows a first-order Markov process. *S_i,t_*:


$$\begin{array}{c} P\left(S_{i,t}=\text{1}|S_{i,t-\text{1}}=\text{0}\right)=p_{\text{onset}} \end{array}$$

$$\begin{array}{c} P\left(S_{i,t}=\text{1}|S_{i,t-\text{1}}=\text{1}\right)=p_{\text{persist}} \end{array}$$

We set $p_{\text{onset}}$=0.03 and $p_{\text{persist}}$=0.80, giving stationary prevalence 0.03/(0.03 + 1 − 0.80)=0.1304. The recorded panel has T=3 and size-dependent initial probabilities of 0.18, 0.15, and 0.12, so its observed prevalence is 0.1564 rather than the stationary value.

#### 4.3.2. Strategy assignment and construction rules.

Conditional on the simulated risk state, the generator assigns one of four strategies with probabilities 0.40, 0.30, 0.20, and 0.10; a new strategy is drawn at a new onset, while persistent risky states retain the prior strategy.

(1) Revenue Suppression reduces true revenue by a uniformly drawn 2%–5% and then recomputes affected flow quantities.
(2) Cost Inflation increases true cost by a uniformly drawn 25%–40% and assigns a strategy-specific invoice-risk range.
(3) Transfer Manipulation adds 20%–35% of pre-shift profit to cost and assigns related-party and transfer-pricing indicators from strategy-specific ranges.

For Revenue Suppression, Cost Inflation, and Transfer Manipulation, profit is recomputed as reported revenue minus reported cost after manipulation, and tax is reset to 25% of positive reported profit, or zero for non-positive profit. Compliant observations retain their baseline simulated effective tax rates, drawn from a normal distribution with mean 0.25 and standard deviation 0.03 and clipped to [0.15, 0.35]. For the Shell Company stress test, tax is set to 10% of reported profit, as described below. These strategy-dependent tax rules create a design-induced tax-rate signature in the relationship between tax and profit.

The four strategies use different scales. Only revenue suppression is restricted to 2%–5% of its baseline. Cost inflation changes true cost by 25%–40%, transfer manipulation adds 20%–35% of pre-shift profit to cost, and the shell-company rule is an overt stress test. Table 4 uses the same notation and ranges as the executable code.

**Table 4:** Typology of Simulated Tax Evasion Strategies.

Risk strategy	Probability conditional on risk	Implemented manipulation	Interpretive role
Revenue Suppression	0.40	revenue=true_revenue×(1−δ), δ~U(0.02,0.05)	Subtle flow manipulation; ratios and VAT gap recomputed
Cost Inflation	0.30	cost=true_cost×(1+κ), κ~U(0.25,0.40)	Large cost and invoice signals
Transfer Manipulation	0.20	cost=cost+profit×γ, γ~U(0.20,0.35)	Large transfer-pricing indicator in Level 2
Shell Company	0.10	asset=100×liability; cost=0.95×revenue; tax=0.10×profit	Deliberately overt stress test

(4) Shell Company is an overt stress-test strategy: assets are set to 100 times liabilities, cost to 95% of revenue, profit to 5% of revenue, and tax to 10% of profit.

### 4.4. Observable signal generation: Measurement noise

The internal simulated states are transformed into observable indicators through a stochastic measurement layer. The distributions below are generator settings, not estimates of measurement error in administrative records.

Value-Added Tax (VAT)-Revenue Discrepancy: VAT sales equal true revenue multiplied by Normal(1,0.10²), with nonnegative clipping; income_gap_ratio then compares VAT sales with reported revenue.


$$\begin{array}{c} R_{i,t}^{\text{VAT}}=R_{i,t}^{\text{true}}\times \left(\text{1}+\epsilon_{\text{vat}}\right),\;\epsilon_{\text{vat}}\sim N\left(\text{0},\;{\text{0}.\text{1}\text{0}}^{\text{2}}\right) \end{array}$$ (11)

Invoice Risk Indicator: the high-risk invoice ratio is drawn from strategy-specific overlapping distributions. Compliant firms use U(0.05,0.30), cost-inflation firms use U(0.20,0.35), and the other risky strategies use U(0.05,0.15). These are construction rules whose realized effects are evaluated in Table 1 and Fig 4, rather than assumed properties of real firms.

**Fig 4:**
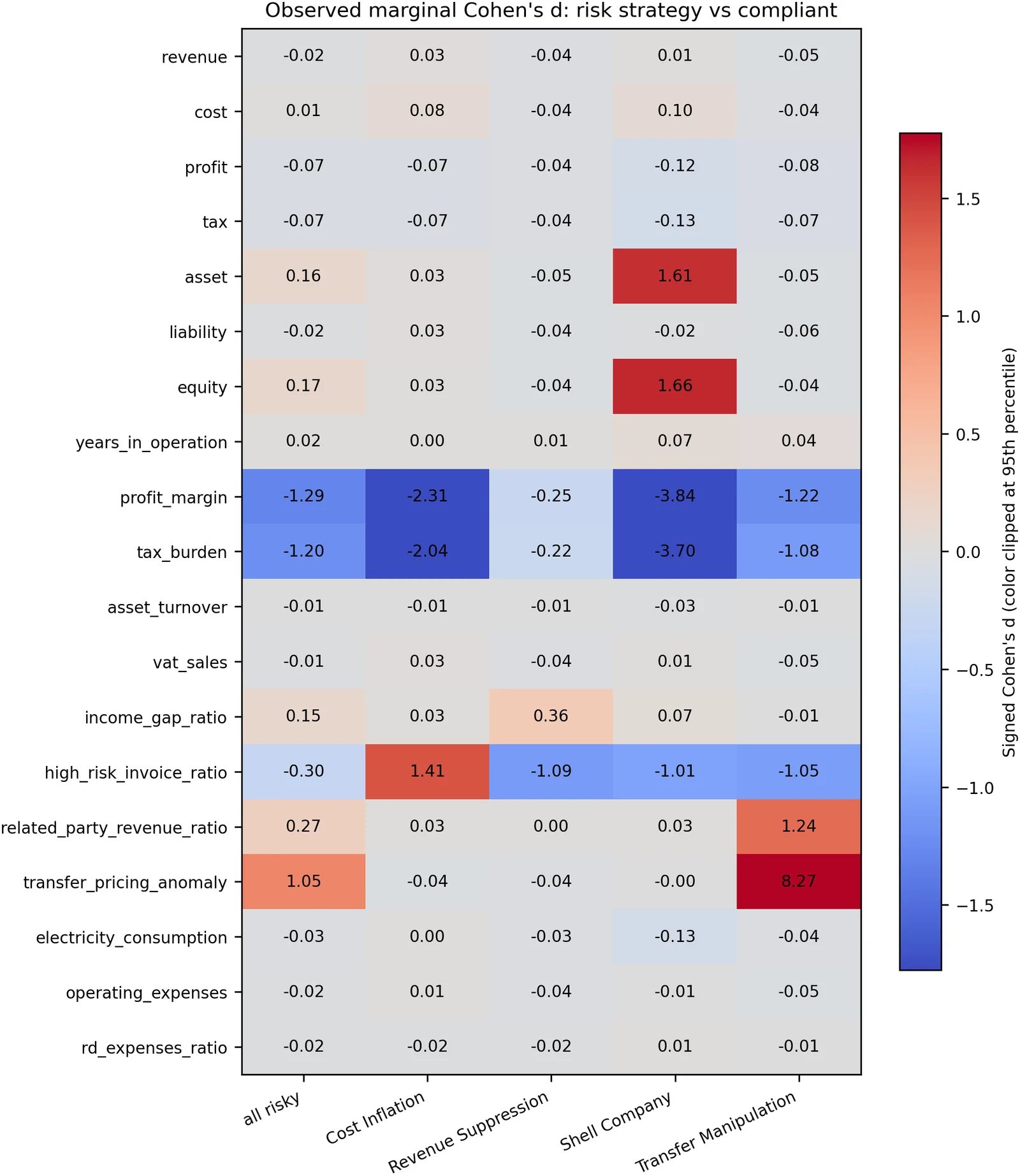
Observed marginal Cohen’s d by risk strategy. Each cell compares the named simulated strategy with compliant observations using the pooled-standard-deviation definition. The all-risk column uses all risky rows. Several features have large effects, including profit margin, tax burden, the cost-inflation invoice indicator, shell-company stocks, and the transfer-pricing anomaly. This figure replaces the original schematic signal-strength threshold claim.

Physical Verification Signal: expected electricity equals true revenue times an industry coefficient. Observed electricity is multiplied by Normal(1,0.30²) and clipped at zero; shell companies instead receive 10%–30% of expected electricity.


$$\begin{array}{c} \text{Ele}{\text{c}}_{i,t}=R_{i,t}^{\text{true}}\times \kappa_{k}\times \left(\text{1}+\epsilon_{\text{elec}}\right),\;\epsilon_{\text{elec}}\sim N\left(\text{0},\;{\text{0}.\text{3}\text{0}}^{\text{2}}\right) \end{array}$$ (12)

where $\kappa_{k}$ denotes the industry-specific electricity intensity coefficient.

These measurement mechanisms can themselves encode risk labels—for example, VAT sales are generated from true revenue while the income-gap denominator uses reported revenue. We therefore evaluate models with and without income_gap_ratio and report Level 1-only results.

### 4.5. Data division: Feature sets

Observable variables are divided into Level 1 financial/reporting and firm-characteristic variables and Level 2 verification or audit-access variables (Table 2). This is a modeling distinction, not a legal determination that any variable is universally public or privacy-free.

Level 1 (financial/reporting variables): simulated statement quantities, derived ratios, and firm characteristics that may be available from filings or registries, depending on jurisdiction.

Level 2 (verification variables): simulated VAT, invoice, related-party, and physical-proxy indicators that generally require tax-system or third-party access.

The partition supports transparent ablation analyses. It does not establish that Level 1 variables are sufficient for real audits or that their use is privacy-neutral.

### 4.6. Implementation, validation design, and reproducibility

TDGM and all revision analyses are implemented in Python with recorded configurations and deterministic seeds. The analytical dataset contains 10,000 firms observed for three years (30,000 rows), with observed risk prevalence 15.64% overall (16.66%, 15.49%, and 14.78% by year). Initial risk probabilities are 0.18, 0.15, and 0.12 for small, medium, and large firms; subsequent onset and persistence probabilities are 0.03 and 0.80, respectively. The implied stationary prevalence for the two-state transition process is 0.03/(0.03 + 1 − 0.80)=0.1304, but the short panel retains the size-dependent initialization effect.

Predictive analyses used five repeated 70/30 group splits by company_id. Thus, all three rows from a firm were assigned wholly to training or test data; the earlier row-stratified design would have placed 95.74% of test firms in the training set. Continuous variables were standardized within each training fold for logistic regression, naive Bayes, and the neural network. The primary neural network used MLPClassifier with one 100-unit ReLU hidden layer, Adam optimization, learning_rate_init = 0.001, alpha = 0.0001, max_iter = 500, and early stopping with a 10% internal training-fold validation fraction and n_iter_no_change = 20. We report mean±SD ROC-AUC, PR-AUC (average precision), F1 at 0.5, Brier score, quantile-bin expected calibration error (ECE), and Precision@Top20%, defined on the held-out test set as the risk prevalence among the highest-scored 20% of observations. For the noise stress test, each numeric held-out feature was multiplied by 1+e independently, where e~N(0,0.15²); training data were unchanged. Prevalence sensitivity used predictions from all five unseen-firm test sets and, within each split, 30 deterministic case-mix resamples at each target prevalence. We also prespecified feature ablations and per-strategy one-versus-compliant analyses.

These analyses are organized as a reusable audit protocol for mechanism-guided synthetic generators: (1) trace active, derived, and legacy parameters from documentation to code; (2) verify hard constraints at the row level; (3) quantify realized—not merely intended—signal strength overall and by mechanism; (4) prevent entity-level leakage in validation; (5) assess discrimination, prevalence-sensitive utility, calibration, ablation, and stress sensitivity; and (6) compare alternative generators under matched inputs while reporting the fidelity cost of constraint repair.

For the matched cross-sectional CTGAN comparison, we selected year 2 so that each of 10,000 firms contributed one row and modeled the same seven continuous financial variables: revenue, cost, profit, tax, assets, liabilities, and equity. SDV CTGAN v1.30.0 used 50 epochs, batch size 500, CPU execution, and seeds 41–43; each run generated 10,000 rows. We evaluated identity validity, the median two-sample Kolmogorov–Smirnov statistic, and Spearman correlation-matrix mean absolute error before and after deterministic projection (profit = revenue−cost; equity = assets−liabilities). The reproducible code, synthetic data, full numerical outputs, and parameter dictionary are listed in the Supporting Information captions.

## 5. Results

The results below are internal validation and stress tests of the synthetic benchmark. They do not estimate real-world evasion prevalence, causal effects, or deployment performance.

### 5.1. Constraint and distributional validation

Table 5 reports a like-for-like cross-sectional comparison between the TDGM reference, raw CTGAN, and CTGAN followed by transparent accounting projection. Temporal autocorrelation is excluded because the single-table CTGAN configuration has no entity-sequence schema.

**Table 5:** Cross-sectional constraint and fidelity comparison between TDGM and CTGAN ^a^.

Metric	TDGM reference	Raw CTGAN, mean±SD	Projected CTGAN, mean±SD
Income-statement identity valid, %	100.0	0.0 ± 0.0	100.0 ± 0.0
Balance-sheet identity valid, %	100.0	0.0 ± 0.0	100.0 ± 0.0
Median marginal KS statistic	0.000	0.152 ± 0.034	0.160 ± 0.048
Spearman correlation-matrix MAE	0.000	0.415 ± 0.011	0.517 ± 0.015

^a^The reference/training data contain one year-2 observation from each of 10,000 firms. CTGAN results are mean±SD across seeds 41–43. Identity validity uses the larger of an absolute tolerance of 1 currency unit and relative tolerance 10^−8^. Lower values are better for KS and correlation MAE. The projected variant deterministically replaces profit and equity after sampling.

Raw CTGAN did not reproduce either row-level identity within tolerance, while deterministic projection restored both to 100%. The projection was not cost-free: median KS increased from 0.152 to 0.160 and Spearman correlation-matrix MAE increased from 0.415 to 0.517. Every sampled profit and equity value changed. These results illustrate a trade-off between constraint-by-construction and post-sampling repair; they do not establish general superiority of TDGM over learned generators.

The previous CTGAN temporal score was removed because independently sampled rows cannot be assigned to pseudo-firms and treated as a panel. TDGM’s separate T=30 run remains only as an internal long-horizon check of the programmed AR(1) mechanism. Detection results use T=3, and temporal adequacy for real audit panels remains untested.

Heavy-tail check. Fig 3 provides an internal check of the programmed Pareto firm-size mechanism. The estimated rank-size slope is −1.046, close to the −1 benchmark used in the design. Because the target was imposed by the simulator, this is an implementation check rather than external validation.

Generated firm sizes (blue points) are shown against the reference scaling used in the design (grey dashed line, slope −1.00); the fitted line has slope −1.046. No claim is made that this fit validates a specific real tax population.

### 5.2. Classifier benchmarking as generator validation

This section measures the predictive structure created by TDGM. It is not a comparative effectiveness study of audit algorithms on real cases.

#### 5.2.1. Experimental setup.

The realized marginal effects are not uniformly below 0.2. Across all risky observations, profit_margin (d=−1.294), tax_burden (d=−1.198), and transfer_pricing_anomaly (d=1.045) have large effects; income_gap_ratio is 0.150 overall but 0.355 for revenue suppression. Table 1 reports all 19 numeric feature effects, and Fig 4 separates them by strategy.

The 30,000 firm-year rows were evaluated using five repeated group splits by firm. Each split assigned 70% of firms to training and 30% to testing, with zero shared firms. The observed prevalence was 15.64%. Classifier metrics in Tables 6 and 7 and the ROC-AUC/PR-AUC entries in Table 8 are reported as means and standard deviations across the five unseen-firm test sets. Table 1 effect sizes use all 30,000 rows; Brier score and ECE entries in Table 8 are five-split means, with complete split-level values in S5 Data.

**Table 6:** Classifier performance under repeated unseen-firm splits ^a^.

Model	ROC-AUC	PR-AUC	F1	Precision@Top20%
Neural network	0.9942 ± 0.0008	0.9710 ± 0.0025	0.9091 ± 0.0045	0.7747 ± 0.0103
XGBoost	0.9714 ± 0.0013	0.8975 ± 0.0034	0.7469 ± 0.0178	0.6827 ± 0.0111
Random forest	0.9328 ± 0.0045	0.8175 ± 0.0123	0.7003 ± 0.0175	0.5943 ± 0.0104
Logistic regression	0.8858 ± 0.0035	0.7620 ± 0.0073	0.6750 ± 0.0172	0.5616 ± 0.0086
Naive Bayes	0.7981 ± 0.0115	0.6754 ± 0.0154	0.5365 ± 0.1133	0.4868 ± 0.0105

^a^Values are mean±SD across five 70/30 group splits by firm. PR-AUC denotes average precision. F1 uses a 0.5 threshold. Precision@Top20% is calculated only on held-out observations. Neural-network results use one 100-unit hidden layer, α=10^−4^, max_iter = 500, and early stopping; 16 units and α=0.1 or 10 are reported only in the controlled sensitivity analysis (S5 Data).

**Table 7:** Strategy-specific XGBoost performance with full and Level 1 feature sets ^a^.

Strategy	Feature set	ROC-AUC	PR-AUC
Revenue Suppression	Full	0.9431 ± 0.0056	0.4740 ± 0.0364
Revenue Suppression	Level 1	0.8802 ± 0.0114	0.3184 ± 0.0432
Cost Inflation	Full	0.9777 ± 0.0013	0.9220 ± 0.0041
Cost Inflation	Level 1	0.9939 ± 0.0009	0.9468 ± 0.0051
Transfer Manipulation	Full	1.0000 ± 0.0000	0.9997 ± 0.0002
Transfer Manipulation	Level 1	0.9793 ± 0.0021	0.7264 ± 0.0294
Shell Company	Full	1.0000 ± 0.0000	1.0000 ± 0.0000
Shell Company	Level 1	1.0000 ± 0.0000	1.0000 ± 0.0000

**Table 8:** XGBoost feature ablations, noise robustness, and calibration ^a^.

Feature/noise scenario	ROC-AUC	PR-AUC	Brier	ECE
Full, 0% noise	0.9714 ± 0.0013	0.8975 ± 0.0034	0.0456	0.0248
Full minus tax and tax_burden	0.9295 ± 0.0022	0.8112 ± 0.0089	—	—
Full minus income_gap_ratio	0.9702 ± 0.0025	0.8934 ± 0.0068	0.0465	0.0254
Level 1 with derived ratios	0.9468 ± 0.0042	0.8278 ± 0.0097	0.0602	0.0329
Level 1 with derived ratios, minus tax and tax_burden	0.8308 ± 0.0064	0.6824 ± 0.0111	—	—
Level 1 raw; no ratios	0.8941 ± 0.0027	0.7435 ± 0.0046	0.0734	0.0262
Full, 15% noise	0.8576 ± 0.0047	0.6691 ± 0.0134	0.1083	0.0780
Level 1, 15% noise	0.7603 ± 0.0046	0.5707 ± 0.0034	0.0989	0.0608

^a^Table 7 uses one-versus-compliant labels and reports means±SD across five unseen-firm test splits. Table 8 uses the aggregate risky label and reports five-split means; ROC-AUC and PR-AUC also show between-split SD. Multiplicative noise is applied only to held-out numeric features. Brier and ECE assess probabilistic calibration; lower values are better. The two tax-feature ablation rows report additional post hoc analyses that jointly exclude tax and tax_burden, with all remaining features retained and no added measurement noise. ROC-AUC and PR-AUC are reported as means ± sample SD across the same five unseen-firm splits. Brier and ECE were not calculated for these analyses and are indicated by em dashes.

Shell companies account for 10% of risky observations and are intentionally overt. Cost inflation and transfer manipulation also create large marginal effects in selected variables. Consequently, aggregate near-ceiling performance cannot be attributed solely to subtle multivariate structure; per-strategy results are reported in Table 7.

#### 5.2.2. Detection results.

Table 1 reports observed effect sizes, and Table 6 compares five prespecified classifiers under the corrected grouped design.

The model ranking changed after correcting the MLP regularization and enforcing grouped validation. The neural network achieved the highest mean ROC-AUC (0.9942) and PR-AUC (0.9710), followed by XGBoost (0.9714 and 0.8975). Logistic regression reached ROC-AUC 0.8858 and PR-AUC 0.7620. These values characterize this simulator and do not establish an algorithm hierarchy for real tax data.

The neural-network sensitivity analysis showed that the original α=10 setting was strongly performance-limiting: relative to α=10^−4^, it reduced full-feature ROC-AUC by about 0.135 and PR-AUC by about 0.249 for the 16-unit architecture. Increasing hidden units from 16 to 100 had much smaller and non-monotonic effects. We therefore removed the prior inference that neural networks lack an appropriate inductive bias based on the over-regularized model.

For XGBoost, the primary grouped estimate was ROC-AUC 0.9714 ± 0.0013 and PR-AUC 0.8975 ± 0.0034. Its Brier score was 0.0456 and quantile-bin ECE was 0.0248. The neural network’s corresponding Brier score and ECE were 0.0217 and 0.0089. These calibration summaries are descriptive because no post-hoc recalibration was fitted.

Because the label and features are generated by the same programmed mechanisms, high discrimination mainly shows that the implementation created recoverable label information. External validity requires evaluation on independently governed or administrative data.

#### 5.2.3. Audit-budget metric.

Precision@Top20% is the fraction of risky observations among the highest-scored 20% of a held-out test set. It is a benchmark ranking metric commonly related to lift and top-decile or top-budget evaluation in prior tax and customs studies [4–6].

In the synthetic test sets, Precision@Top20% was 0.7747 ± 0.0103 for the neural network, 0.6827 ± 0.0111 for XGBoost, and 0.5616 ± 0.0086 for logistic regression. These values are illustrative consequences of the simulated prevalence, label rules, and budget fraction; they do not imply numbers of real evaders recovered per 100 audits.

### 5.3. Strategy, feature-accessibility, and noise analyses

Table 7 separates the four programmed risk strategies, and Table 8 reports feature ablations, noise stress tests, and calibration metrics for XGBoost.

#### 5.3.1. Strategy-specific behavior.

Revenue suppression was the most difficult strategy: full-feature XGBoost achieved ROC-AUC 0.9431 and PR-AUC 0.4740, falling to 0.8802 and 0.3184 with Level 1 features. In contrast, cost inflation, transfer manipulation, and shell companies produced high discrimination because their rules create large or overt signals.

For aggregate risk, removing income_gap_ratio changed XGBoost ROC-AUC from 0.9714 to 0.9702 and PR-AUC from 0.8975 to 0.8934. Thus, this single verification variable is not solely responsible for the aggregate score. However, Level 2 variables materially improve the revenue-suppression result, and their construction remains a potential source of label proximity.

#### 5.3.2. Feature-set ablations and measurement noise.

Using Level 1 variables including derived ratios yielded ROC-AUC 0.9468 and PR-AUC 0.8278. Removing both Level 2 variables and derived ratios reduced performance to 0.8941 and 0.7435. The difference shows that derived ratios carry design-induced signal; it does not demonstrate that Level 1 inputs are sufficient for real audit decisions.

A code-informed post hoc diagnostic identified a design-induced tax-rate signature in the supplied synthetic panel. The tax-to-profit relationship, together with a non-positive-profit condition, allowed recovery of the programmed risk labels in this panel. In an additional post hoc sensitivity analysis, we jointly excluded tax and tax_burden using the same five prespecified unseen-firm splits and XGBoost specification, leaving all remaining feature values unchanged. For the full feature set, mean ROC-AUC decreased from 0.9714 to 0.9295 and mean PR-AUC (average precision) from 0.8975 to 0.8112. For Level 1 including derived ratios, the corresponding means decreased from 0.9468 to 0.8308 and from 0.8278 to 0.6824, respectively. These are arithmetic means across the five splits. This ablation demonstrates the contribution of tax-related inputs to the fitted models but does not isolate the fixed-rate rule from other information contained in those inputs (Table 8).

With 15% multiplicative noise, full-feature XGBoost fell to ROC-AUC 0.8576 ± 0.0047 and PR-AUC 0.6691 ± 0.0134; Level 1 fell to 0.7603 ± 0.0046 and 0.5707 ± 0.0034. Noise therefore weakens both discrimination and calibration rather than supporting a general claim of noise immunity.

#### 5.3.3. Calibration.

The full-feature baseline had Brier score 0.0456 and ECE 0.0248; under 15% noise these worsened to 0.1083 and 0.0780. Level 1 baseline values were 0.0602 and 0.0329, worsening under noise to 0.0989 and 0.0608. Calibration plots and bin-level values are supplied in S5 Data. Thresholds would require independent recalibration before any operational use.

### 5.4. Sensitivity analysis

#### 5.4.1. Observed marginal effect sizes.

Fig 4 reports Cohen’s d calculated directly from the 30,000-row dataset for all risky observations and separately for each strategy. The signs indicate the direction of the risky-minus-compliant difference; color limits are clipped only for display, while cell annotations show the underlying values.

The revenue-suppression strategy is mixed: most raw monetary features have negligible marginal effects, but income_gap_ratio has d=0.355 and high_risk_invoice_ratio has d=−1.086 because the code assigns different invoice ranges to non-cost-inflation risky firms. Cost inflation, transfer manipulation, and shell-company rules generate multiple large effects.

Accordingly, the revised manuscript does not define a universal ‘grey-zone threshold’ at ROC-AUC 0.975 and does not claim that all simulated evasion is locally indistinguishable. The effect-size table describes the observed signal strength within this generator.

#### 5.4.2. Prevalence sensitivity.

To examine metric behavior under class imbalance, held-out predictions from all five unseen-firm test splits were resampled to target prevalences from 1% to 20%. Within each split and target prevalence, 30 deterministic case-mix resamples were averaged; uncertainty was then summarized across the five split-level means. The exercise changes the case mix of the simulated test set; it is not evidence of transportability across jurisdictions.

As shown in Fig 5, XGBoost ROC-AUC remained near 0.971 across target prevalences, but PR-AUC changed substantially: 0.670 at 1%, 0.797 at 5%, 0.894 at 15%, and 0.916 at 20%. Precision@Top20% likewise rose from 0.049 at 1% to 0.810 at 20%.

**Fig 5:**
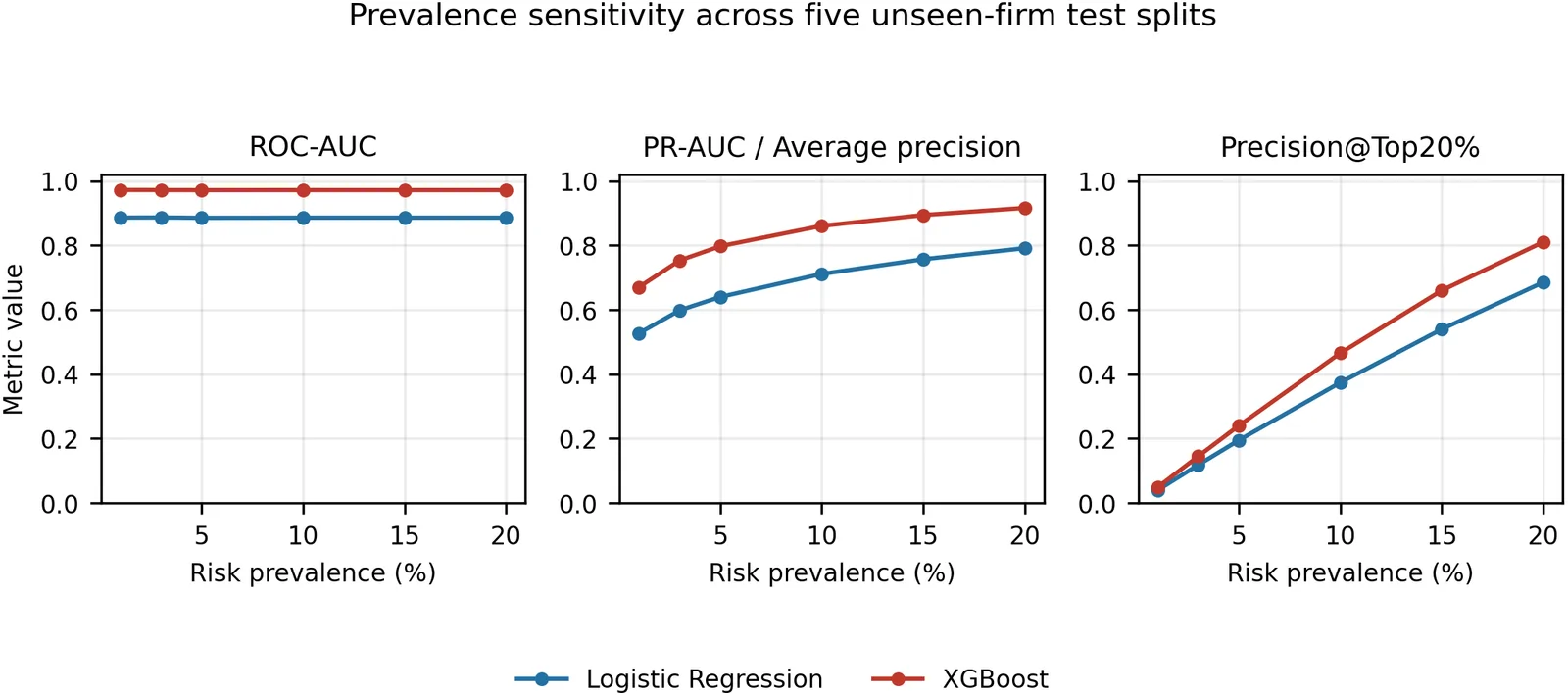
Prevalence sensitivity across five repeated unseen-firm test splits. ROC-AUC, PR-AUC (average precision), and Precision@Top20% are shown for logistic regression and XGBoost after resampling to target prevalences of 1%, 3%, 5%, 10%, 15%, and 20%. Each plotted value is the mean of five split-level estimates, each based on 30 deterministic case-mix resamples. Stable ROC-AUC does not imply stable precision or calibration under class imbalance.

At the 1% target prevalence, XGBoost mean predicted risk was 0.0624 and Brier score was 0.0136; logistic regression mean predicted risk was 0.0866 and Brier score was 0.0218. Predictions were intentionally not recalibrated after case-mix resampling, so the difference between target prevalence and mean predicted risk illustrates why transported probabilities require recalibration. Under rare outcomes, PR-AUC and audit-budget precision are more informative than ROC-AUC alone.

These results show a standard metric property within the synthetic test set: rank-based ROC-AUC can remain stable while prevalence-sensitive utility changes sharply. They do not support deployment without local prevalence estimation, recalibration, and external validation.

## 6. Discussion

### 6.1. What the benchmark validates—and does not validate

The revised experiments show that TDGM creates strongly recoverable signals, but the ranking of classifiers depends on a previously non-default neural-network regularization choice. With α=10^−4^, the MLP outperforms XGBoost on this benchmark. This reverses the original claim and demonstrates why benchmark conclusions must be separated from claims about model classes in real tax data.

The generator combines subtle and overt mechanisms rather than one uniformly concealed regime. Consequently, its aggregate score reflects a mixture of strategy prevalence and signal strengths. The per-strategy and effect-size analyses are more informative than a single ‘nonlinear advantage’ narrative.

The analytical arguments in S1 Appendix are now presented as design implications: if rules create nonlinear interactions or flow–stock mismatches, flexible models may exploit them. The numerical results verify that the code produces learnable structure; they do not independently validate those assumptions in observed tax systems.

Taken together, parameter tracing, constraint verification, realized-signal analysis, entity-level leakage control, utility and calibration stress testing, and matched comparator repair define a reusable audit protocol for mechanism-guided synthetic generators. Its appropriate use is controlled software testing, sensitivity analysis, and reproducible benchmarking. Model selection for enforcement would require independently collected labels, jurisdiction-specific sampling, calibration, fairness assessment, and prospective evaluation.

### 6.2. Feature accessibility and design-induced structural signals

Level 1 derived features retain much of the aggregate synthetic performance, but raw Level 1 variables perform materially worse. This difference is consistent with the simulator’s construction because profit margin, tax burden, and asset turnover are deterministic transformations of variables changed by the risk rules.

The term ‘structural shadow’ is retained as a design concept: modifying selected flows after stocks have been generated can create detectable ratios. In this paper it is a programmed mechanism and analytical consequence, not an empirically established behavioral law.

Level 2 variables add direct verification signals and improve some strategy-specific results, particularly revenue suppression. The ablation excluding income_gap_ratio shows that no single near-oracle feature explains the aggregate XGBoost score, but the entire set of generated verification variables remains coupled to the risk mechanism.

These findings motivate a testable future hypothesis—that carefully chosen filing-derived ratios might support first-stage screening—but do not demonstrate audit effectiveness, privacy compliance, or legal suitability. Those questions depend on the jurisdiction, access rules, missingness, measurement error, and selection processes in real records.

We use the neutral label ‘Level 1 financial/reporting variables.’ Public availability and sensitivity differ across jurisdictions, and even disclosed financial statements can contain commercially sensitive information. No privacy guarantee is inferred from synthetic predictive performance.

### 6.3. Relation to audit-targeting literature and operational metrics

Top-budget precision is useful for describing rankings under constrained review capacity, but it is not new to tax analytics. Prior studies use lift, top-decile targeting, recovered revenue under a fixed audit share, and policy-allocation simulations [4–6]. We use Precision@Top20% as one comparable benchmark metric and report it with PR-AUC.

Any translation from synthetic precision to numbers of real cases found is conditional arithmetic, not a policy estimate. Real audit yield depends on case prevalence, label verification, selection bias, deterrence, audit cost, taxpayer responses, and calibration drift.

The contribution of the detection exercise is diagnostic: it reveals what the generator makes easy or difficult, identifies leakage and regularization sensitivity, and supplies outputs against which future methods can be reproduced. It does not justify a particular enforcement workflow.

### 6.4. Limitations and future directions

TDGM remains a synthetic benchmark with important limitations. These limitations constrain both the generator and every predictive result derived from it.

Synthetic-only evidence and circularity. No administrative audit records, external financial statements, or independently labeled tax cases were used. The mechanisms generate both predictors and labels, so high discrimination is an internal property of the simulation. External realism, fairness, audit yield, and policy impact remain unknown.

Parameterization and dynamics. Pareto, AR(1), industry, Markov, and manipulation parameters are scenario assumptions assembled from code defaults and limited literature rather than jointly calibrated to one target population. AR(1) dynamics omit macroeconomic breaks, common shocks, entry and exit, and heterogeneous persistence. The T=30 run checks the programmed process only; the detection dataset has T=3.

Static strategies and feature construction. Strategy probabilities and manipulation ranges are fixed, and firms do not adapt to audits or model deployment. Several variables are generated directly from true states or strategy-specific distributions, creating strong effects and possible label proximity. Future versions should model adaptive behavior and validate features against independent processes.

The post hoc tax-rate diagnostic further shows that tax-related inputs can encode programmed risk structure even at Level 1. The diagnostic characterizes the supplied synthetic panel and is not an independently validated predictive result. Accordingly, both the strong discrimination and the changes observed under tax-feature ablation should be interpreted within this synthetic design and do not establish generalizable tax-risk discrimination.

Baseline and generalization limits. The CTGAN experiment is a cross-sectional comparison with one model family, three seeds, and modest training settings. It cannot represent panel dependence, and the TDGM training reference is itself synthetic. Comparisons with constraint-aware, causal, copula, and longitudinal generators remain future work. Any operational use would additionally require local governance, privacy review, representative labels, recalibration, and prospective validation.

## 7. Conclusion

This study presents TDGM as a transparent mechanism-guided simulator for synthetic tax-risk benchmarking. The revised analysis verifies accounting constraints, documents realized signal strengths, eliminates firm-level train/test leakage, reports PR-AUC and calibration, and tests feature, noise, prevalence, and MLP-regularization sensitivity.

### 7.1. Framework contribution

The framework demonstrates one practical benefit of constraint-by-construction simulation: specified row-level identities can be guaranteed and audited directly. The matched cross-sectional CTGAN comparison shows that deterministic post-sampling projection can also guarantee those identities, but with measurable changes to distributional and dependence fidelity.

This comparison is not a universal contest between mechanistic and learned generators. It isolates a design trade-off under one cross-sectional task and makes the assumptions and repair operation reproducible.

### 7.2. Internal validation findings

The benchmark does not support the original universal low-effect or XGBoost-superiority claims. Realized effects are heterogeneous, and a conventionally regularized MLP achieved the strongest mean discrimination. XGBoost remained useful for transparent ablation and stress tests, with ROC-AUC 0.9714 and PR-AUC 0.8975 under unseen-firm splits.

Level 1 derived variables retained substantial synthetic performance, but raw Level 1 variables and revenue-suppression-only analyses were materially weaker. These differences identify where the programmed features carry signal.

Prevalence resampling further showed that stable ROC-AUC can coexist with large changes in PR-AUC and Precision@Top20%. Reporting prevalence-sensitive and calibration metrics is therefore essential even within a simulation benchmark.

### 7.3. Scope and future validation

The analytical flow–stock argument is best understood as a design implication of TDGM: changing flows while anchoring stocks creates ratio distortions. Whether comparable patterns occur and remain useful in real filings is an empirical question for future research.

No claim of cross-jurisdictional invariance or deployment without recalibration is made. Future work should use independent administrative or secure-enclave data, model adaptive strategies and macroeconomic shocks, compare longitudinal generators, and evaluate calibration, fairness, audit costs, and prospective utility.

The contribution is therefore a reproducible and inspectable simulation artifact. Its value lies in enabling controlled tests and exposing assumptions—not in substituting synthetic validation for evidence about real taxpayers.

## Supporting information

S1 File Reproducible source code and execution instructions. Archive containing the TDGM generator, matched cross-sectional CTGAN comparison, grouped revision analysis, MLP sensitivity analysis, and figure-generation scripts.
(ZIP)

S1 Data Full TDGM synthetic panel. CSV file containing 30,000 firm-year observations from 10,000 simulated firms over three recorded years. The variable years_in_operation is a computer-generated firm attribute and is not a person’s age; all identifiers and other attributes are synthetic.
(CSV)

S2 Data Signal-strength sensitivity outputs used in the original workflow and retained for reproducibility. (CSV)

S3 Data Prevalence-sensitivity source data supporting Fig 5. CSV summary of logistic-regression and XGBoost metrics across five unseen-firm splits, with 30 deterministic resamples per target prevalence within each split. (CSV)

S4 Data Matched cross-sectional CTGAN comparison results. Seed-level and summary CSV/JSON outputs for raw and accounting-projected CTGAN.
(ZIP)

S5 DataRevision analysis results.Workbook containing grouped model metrics, PR-AUC, calibration, Cohen’s d, strategy-specific results, ablations, noise stress tests, prevalence sensitivity, and reproducibility checks.(XLSX)

S6 Data TDGM parameter dictionary. Workbook mapping manuscript assumptions to active code parameters, observed checks, legacy unused fields, and implemented manuscript revisions.
(XLSX)

S1 Appendix Analytical design implications and model hyperparameter settings [25]. (DOCX)
