# Supplementary material for: A mechanism-guided simulation framework for synthetic tax-risk data: Constraint validation and reproducible benchmarking
Source: PLoS One. 2026 Oct 1;21(10):e0353137. doi: 10.1371/journal.pone.0353137 (PMC13630321; doi:10.1371/journal.pone.0353137)
Supplement: Supplementary file 1 [file pone.0353137.s008.docx]

S8 Appendix

Analytical design implications and model hyperparameter settings

**Appendix A. Analytical design implications**

**A.1. Implication of overlapping simulated distributions**

Design implication 1. If two simulated class-conditional distributions overlap, no deterministic linear hyperplane perfectly separates all observations. This statement describes geometry under the assumed distributions; it does not imply that a nonlinear model must outperform logistic regression in every finite sample.

Reasoning for design implication 1.

Let $X$ denote the realized feature vector and let the two simulated classes have class-conditional distributions P0 and P1. Their degree of overlap is produced by the generator and must be measured from the generated data.

Linear separability concerns the geometry of the full feature representation, not visual overlap in a two-dimensional PCA projection. Convex-hull criteria provide one formal characterization of finite-sample linear separability.[22] However, projected overlap does not establish the shape of a population decision boundary.

Accordingly, TDGM does not assert that its risk classes necessarily require a nonlinear classifier. Linear and flexible models are compared under identical grouped splits, and any observed difference depends on the realized features, model specification, tuning, and regularization.

Overlap can create classification error, and flexible models can represent a broader class of boundaries, but neither fact establishes that one model family must dominate on this benchmark or on real tax data.

**A.2. Implication of flow–stock anchoring**

Design implication 2. Under the simulator’s assumption that a flow variable changes while its stock anchor is held fixed, any ratio combining those quantities changes algebraically. This is a consequence of the programmed rule, not evidence that real firms cannot jointly alter multiple accounts.

Reasoning for design implication 2.

Step 1: Let true revenue be $R$ and true assets be $A$. Revenue suppression sets reported revenue to $R$(1−$\delta$), where 0<$\delta$<1. Assets remain unchanged because the current generator creates stocks before applying this flow manipulation.

Step 2: True asset turnover is $R/A$, whereas reported asset turnover is $R(1-\delta)/A$. The difference is $-\delta R/A$, so the ratio changes whenever $\delta>0$ and $A$ is nonzero. This is a direct algebraic consequence of the programmed ordering, not a new theorem about firm behavior.

The same reasoning applies only to ratios that combine a manipulated quantity with another quantity that the generator leaves unchanged. Its magnitude and predictive usefulness are empirical properties of the generated dataset.

Step 3: The current generator does not implement coordinated stock adjustment after flow manipulation. Other simulators or real accounting processes could alter multiple accounts jointly, so this appendix establishes a code-induced ratio change rather than operational infeasibility.

Conclusion: within TDGM, the ‘structural shadow’ is an expected feature-construction effect. Its existence and usefulness outside the simulator require independent data.

**Appendix B. Model Hyperparameter Settings**

Primary model configurations were prespecified for the revision analysis. The neural network uses scikit-learn MLPClassifier with one 100-unit ReLU hidden layer, Adam optimization, learning_rate_init=0.001, $\alpha$=10⁻⁴, max_iter=500, early_stopping=True, validation_fraction=0.10, and n_iter_no_change=20. A separate controlled sensitivity analysis varies hidden units (16, 100) and $\alpha$ (10⁻⁴, 0.1, 10) under the same five group splits while holding the remaining sensitivity settings fixed. We do not describe $\alpha$=10 as a default setting.

**Table B.1. Hyperparameter Configurations**

| **Model** | **Primary hyperparameters** |
| --- | --- |
| Logistic regression | penalty='l2'; C=1.0; solver='lbfgs'; max_iter=2000 |
| Neural network | hidden_layer_sizes=(100,); ReLU; Adam; learning_rate_init=0.001; alpha=0.0001; max_iter=500; early_stopping=True; validation_fraction=0.10; n_iter_no_change=20 |
| Random forest | n_estimators=100; max_depth=10; random_state recorded by split |
| XGBoost | n_estimators=100; learning_rate=0.1; max_depth=6; eval_metric='logloss' |
| Naive Bayes | GaussianNB; var_smoothing=1e−9 |
